# Thermodynamics of Damaged DNA Binding and Catalysis by Human AP Endonuclease 1

**Published:** 2016

**Authors:** A. D. Miroshnikova, A. A. Kuznetsova, N. A. Kuznetsov, O. S. Fedorova

**Affiliations:** Institute of Chemical Biology and Fundamental Medicine, Siberian Branch of the Russian Academy of Sciences. Prosp. Acad. Lavrent’eva, 8, Novosibirsk, 630090, Russia;; Department of Natural Sciences, Novosibirsk State University, Pirogova St., 2, Novosibirsk, 630090 , Russia.

**Keywords:** thermodynamics, pre-steady-state kinetics, kinetic mechanism, apurinic/apyrimidinic site, human AP endonuclease

## Abstract

Apurinic/apyrimidinic (AP) endonucleases play an important role in DNA repair and initiation of AP site elimination. One of the most topical problems in the field of DNA repair is to understand the mechanism of the enzymatic process involving the human enzyme APE1 that provides recognition of AP sites and efficient cleavage of the 5’-phosphodiester bond. In this study, a thermodynamic analysis of the interaction between APE1 and a DNA substrate containing a stable AP site analog lacking the C1’ hydroxyl group (F site) was performed. Based on stopped-flow kinetic data at different temperatures, the steps of DNA binding, catalysis, and DNA product release were characterized. The changes in the standard Gibbs energy, enthalpy, and entropy of sequential specific steps of the repair process were determined. The thermodynamic analysis of the data suggests that the initial step of the DNA substrate binding includes formation of non-specific contacts between the enzyme binding surface and DNA, as well as insertion of the amino acid residues Arg177 and Met270 into the duplex, which results in the removal of “crystalline” water molecules from DNA grooves. The second binding step involves the F site flipping-out process and formation of specific contacts between the enzyme active site and the everted 5’-phosphate-2’-deoxyribose residue. It was shown that non-specific interactions between the binding surfaces of the enzyme and DNA provide the main contribution into the thermodynamic parameters of the DNA product release step.

## INTRODUCTION

Ones of the most frequent DNA damages are apurinic/ apyrimidinic sites (AP sites) [1, 2] that are formed in DNA during spontaneous or DNA glycosylase-catalyzed hydrolysis of N-glycosidic bonds [3]. Every day, up to 10,000 AP sites may form in the human cell. The high mutagenicity of AP sites is related to both the lack of an encoding nitrogenous base and the increased ability of AP sites to cause nicks in the DNA ribose phosphate backbone.

The key enzyme of the base excision repair (BER) system is human apurinic/apyrimidinic endonuclease 1 (APE1) that is responsible for recognition and initiation of removal of AP sites in DNA [4, 5]. Its major physiological function is hydrolysis of the DNA phosphodiester bond located upstream of the AP site, which results in a ribose phosphate backbone breakage to form chain fragments containing a 3’-hydroxyl group and 2’-deoxyribose 5’-phosphate [6, 7].

**Fig. 1:**
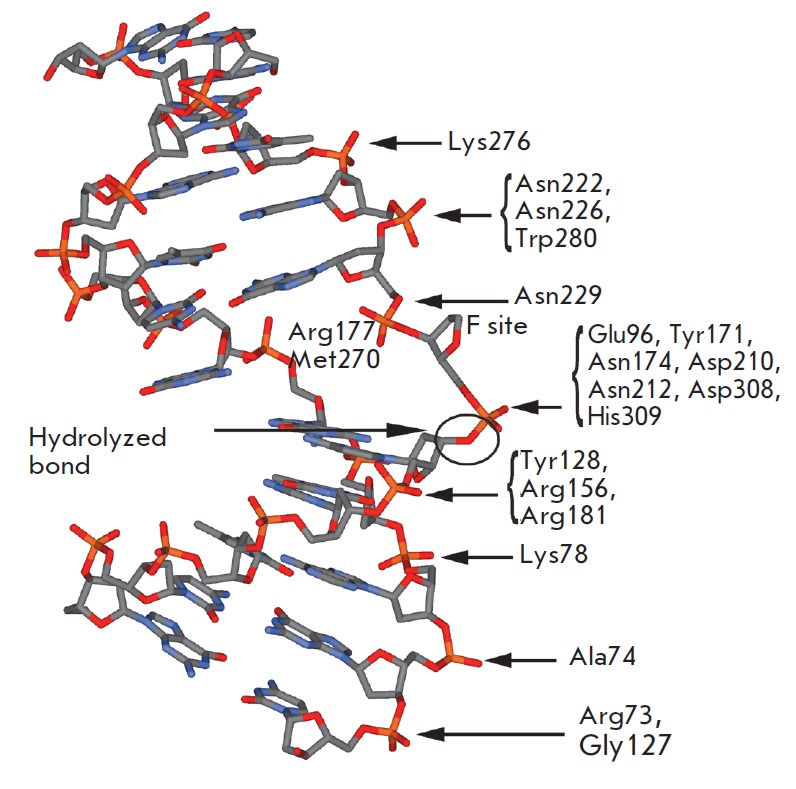
Schematic representation of contacts in the complex between APE1 and DNA containing the F site (PDB ID 1DE8 [11]).

An analysis of the crystal structures of the free APE1 enzyme [8-10] and APE1-DNA covalent complexes [11-13] showed that catalysis in the APE1-DNA complex requires contacts whose formation leads to flipping of an AP site out of the double helix. *Figure 1* presents a scheme of the contacts in the enzymesubstrate complex between APE1 and DNA containing the F site lacking an OH-group in the C1’ position of deoxyribose (PDB ID 1DE8). It is seen that enzyme amino acid residues interact preferentially with one of the duplex strands to form usually hydrogen bonds and electrostatic contacts between DNA phosphate groups and amino acid side chains and also amide groups of peptide bonds of the protein. The enzyme active site is formed by Asp308, His309, Glu96, Asp210, Tyr171, Asn212, and Asn174 residues. The flipped out AP site conformation is stabilized by Met270 and Arg177 residues. Met270 is embedded into the DNA minor groove, thereby displacing the base opposite to the AP site. The Arg177 residue is inserted on the DNA major groove side and forms a hydrogen bond with a phosphate group located downstream of the AP site. In the enzyme-substrate complex, which is in a catalytically competent state, a phosphate residue located upstream of the AP site is coordinated by Asn174, Asn212, and His309 residues. The catalytic reaction begins with the nucleophilic attack of a water molecule that is coordinated, directly or indirectly through a Mg^2+^ ion, by Asp210 on a 5’-phosphate group [11, 13].

Previously, a stopped-flow technique with detection of changes in the fluorescence intensity of enzyme tryptophans [14, 15] and 2-aminopurines located downstream and upstream of the AP site [16] was used to elucidate the kinetic mechanism of interaction between APE1 and DNA substrates (*Scheme*). DNA duplexes containing a native AP site or its analog (F site) without an OH-group in the C1’ position of deoxyribose were used as substrates. The interaction between APE1 and substrates was shown to include at least two steps of DNA binding and AP site recognition that lead to the formation of a catalytically competent complex. An irreversible step of catalytic hydrolysis of a 5’-phosphodiester bond of the AP site occurs in this complex. The last step of the kinetic mechanism describes equilibrium dissociation of the enzyme-product complex.

*Scheme*. The kinetic mechanism of interaction between APE1 and a DNA substrate

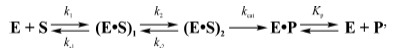



where E is the enzyme; S is the substrate; (E•S)_1_ and (E•S)_2_ are enzyme-substrate complexes; P is a product of the enzyme-catalized reaction; (E•P) is the enzyme- product complex; *k_i_*and *k_–i_*are rate constants of the forward and reverse reactions of equilibrium steps;* k_cat_*is the rate constant of the catalytic step; and *K*_p_ is the equilibrium dissociation constant of the EP complex.

**Fig. 2:**
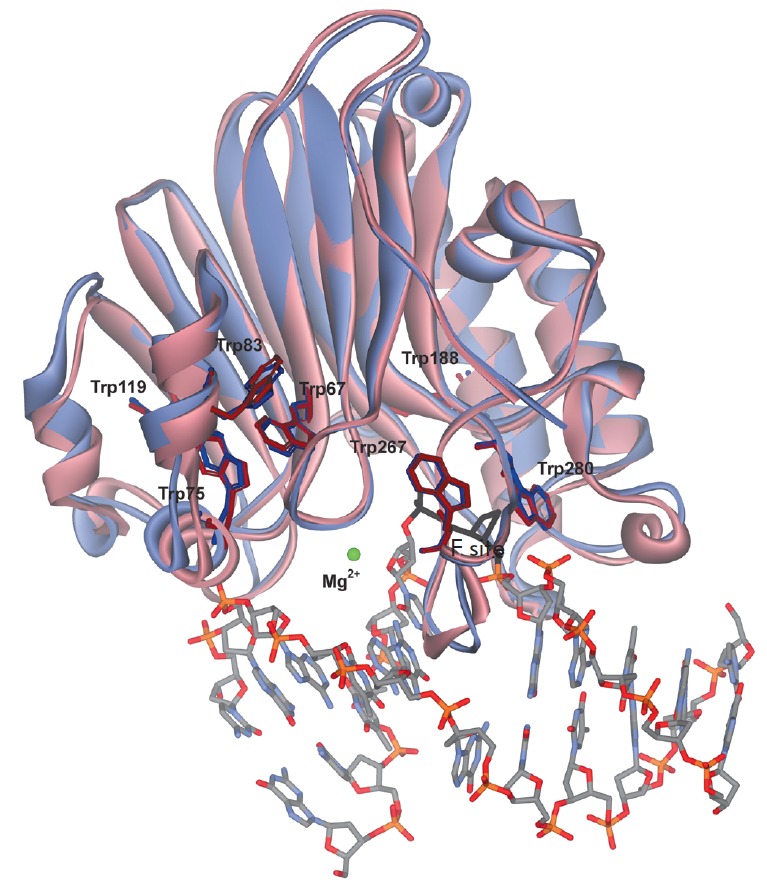
Overall structures of free APE1 (pink, PDB ID 4LND) and APE1 associated with damaged DNA (violet, PDB ID 1DE8).

According to the X-ray data, DNA binding leads only to minor structural rearrangements in APE1 (*Fig. 2*). Comparison of the structures of free APE1 (PDB ID 4LND) and a complex between APE1 and DNA containing the F site (PDB ID 1DE8) demonstrates that one of the seven tryptophan residues of the enzyme molecule, Trp280, is located in the DNAbinding site and forms a hydrogen bond with a DNA phosphate group. Therefore, the observed changes in Trp fluorescence are likely related to enzyme conformational changes in the Trp280 region.

The aim of this work was to determine the thermodynamic parameters of the APE1 conformational rearrangements associated with specific recognition of a damaged DNA site and the catalytic step of the enzymatic reaction during base excision repair based on the kinetic data of the enzymatic process at different temperatures. This approach made it possible to determine the thermodynamic parameters for steps of formation of the catalytically active enzyme form, including an intermediate enzyme-substrate complex, in contrast to the data [17] obtained previously for an inactive form of APE1.

## MATERIALS AND METHODS

**Oligodeoxyribonucleotides**

Oligonucleotides were purified by HPLC on an ion exchange column (PRP-X500 Hamilton Company, 3.9 × 300 mm, 12–30 μm particle size) and subsequent reverse phase chromatography (Nucleoprep 100-20 C18, 10 × 250 mm, Macherey-Nagel, Germany). Oligonucleotide purity was evaluated using 20% denaturing polyacrylamide gel electrophoresis (PAGE). The oligonucleotide concentration was measured by absorbance of solutions at 260 nm in their electronic absorption spectra and calculated according to the Lambert-Bouguer- Beer law based on a molar extinction coefficient determined using the nearest-neighbor approximation [17]. A DNA substrate of the APE1 enzyme (F substrate) was a 17-mer duplex consisting of oligodeoxyribonucleotides

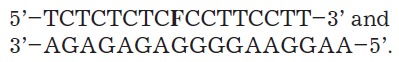



**APE1 enzyme**

The APE1 enzyme was isolated from *Escherichia coli* Rosetta 2 cells transformed with the plasmid pET11a carrying the human AP endonuclease gene. The *E. coli* Rosetta 2 cell culture was grown in a LB medium (1 L) containing 50 μg/mL of ampicillin at 37°C to an optical density of 0.6–0.7 at 600 nm. Then, the temperature was lowered to 20°C, and transcription was induced by adding isopropyl-β-*D*-thiogalactopyranoside to a final concentration of 0.2 mM. After induction, the cell culture was incubated for 16 h. Then, the cells were pelleted by centrifugation (12,000 rpm, 10 min), and a cell suspension was prepared in 30 mL of buffer I (20 mM HEPES-NaOH, pH 7.8) containing 40 mM NaCl. Cells were lysed using a French-press. All subsequent procedures were performed at 4°C. The cell lysate was centrifuged (30,000 rpm, 40 min), and the supernatant was loaded onto column I (Q-Sepharose Fast Flow, Amersham Biosciences, Sweden) and washed with buffer I (20 mM HEPES-NaOH, pH 7.8) containing 40 mM NaCl. Fractions containing the APE1 protein were collected and loaded onto column II (HiTrap-Heparin™, Amersham Biosciences, Sweden). Chromatography was performed in buffer I with a linear gradient of 40 → 600 mM of NaCl; the solution’s absorbance was detected at 280 nm. The APE1 protein purity was determined by gel electrophoresis. Fractions containing the APE1 protein were dialyzed in buffer II (20 mM HEPES-NaOH, 1 mM EDTA, 1 mM dithiothreitol, 250 mM NaCl, 50% glycerol, pH 7.5) and stored at –20°C. The enzyme concentration was calculated using protein absorbance values at 280 nm and a molar extinction coefficient of 56,818 M^–1^ cm^–1^ [19].

**Stopped-flow kinetic measurements**

Kinetic fluorescence curves were acquired using a SX20 stopped-flow spectrometer (Applied Photophysics, UK). The fluorescence excitation wavelength was 290 nm. Fluorescence was recorded at wavelengths longer than 320 nm (Schott filter WG 320). Since the APE1 molecule contains 7 Trp residues and 11 Tyr residues, more than 90% of the detected protein fluorescence intensity was due to Trp fluorescence under the experimental conditions used. The instrument dead time was 1.1 ms, and the maximum signal acquisition time was 200 s. All experiments were performed in a buffer solution simulating BER conditions (50 mM Tris- HCl, 50 mM KCl, 5 mM MgCl_2_, 1 mM dithiothreitol, 7% glycerol, pH 7.5) at 10–37°C. Each kinetic curve was an average of at least three experimental curves.

**Analysis of the hydrolysis extent of the 5’-phosphodiester bond at the AP site**

The dependence of the hydrolysis extent of the 5’-phosphodiester bond at the AP site on time was studied by mixing enzyme and ^32^P-labeled substrate solutions. The label was attached to the 5’-end of an F-containing oligonucleotide using T4 polynucleotide kinase (SibEnzyme, Novosibirsk) and [γ-^32^P] ATP (BIOSAN, Novosibirsk) according to [20, 21]. Further, 2 μL aliquots were taken from the reaction mixture and transferred to prepared test tubes containing 3 μL of a 7 M urea solution, 0.1% bromophenol blue, and 0.1% xylene cyanol FF. PAGE was carried out at 50 V/cm. Gel was autoradiographed on an Agfa CP-BU New Xray film (Agfa-Geveart, Belgium) at –20°C for 12–60 h.

**Analysis of kinetic curves**

To calculate the rate constants of conformational transitions, a number of kinetic curves for different substrate concentrations at different temperatures were obtained. Detection was carried out under conditions appropriate for one enzyme turnover, i.e. at enzyme and substrate concentrations of the same order. DynaFit software (BioKin, USA) [22] was used to determine the minimum kinetic scheme describing the enzyme- substrate interaction and to calculate the rate constants of elementary steps of the reaction. Quantitative processing of experimental data was conducted by optimizing the parameters included in the kinetic schemes as described previously [23-25].

The obtained values of rate constants of individual reaction steps were used to calculate the equilibrium constants (*K*_i_) for the steps (*k_i_*/*k_–i_*, where *i *is the step number) at different temperatures. Standard thermodynamic parameters of the *i*-th equilibrium step were determined using the van’t Hoff equation (1) [26, 27]

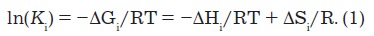



The ln(*K*_i_) vs 1/T dependences were linear.

An analysis of the temperature dependence of the reaction rate constant *k*_cat_ using the Eyring equation (2) provided the standard activation enthalpy (ΔH^o,‡^) and standard activation entropy (ΔS^o,‡^) of the transition state [26]

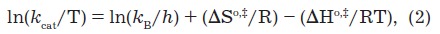



where *k*_B_ and *h *are the Boltzmann and Planck constants, respectively; R is the gas constant; and T is absolute temperature in degrees Kelvin.

## RESULTS AND DISCUSSION

**Fig. 3:**
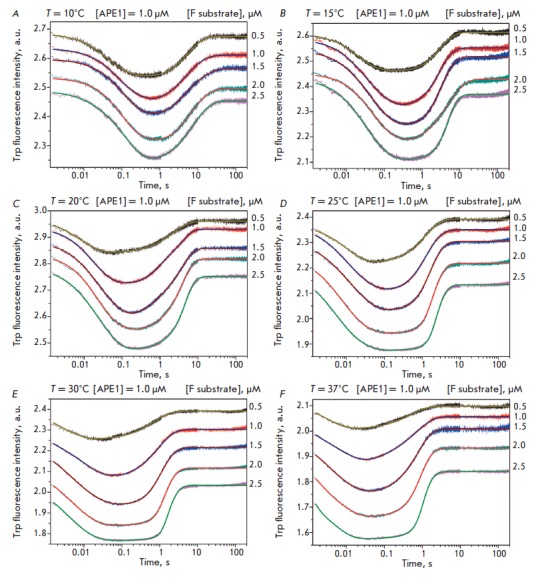
Changes in the Trp fluorescence intensity during interaction between APE1 and the F substrate at 10°C (A), 15°C (B), 20°C (C), 25°C (D), 30°C (E), and 37°C (F). Experimental curves were smoothed using the kinetic model (*Scheme*). The F substrate concentration was varied from 0.5 to 2.5 μM; the APE1 concentration was 1.0 μM.

**Fig. 4:**
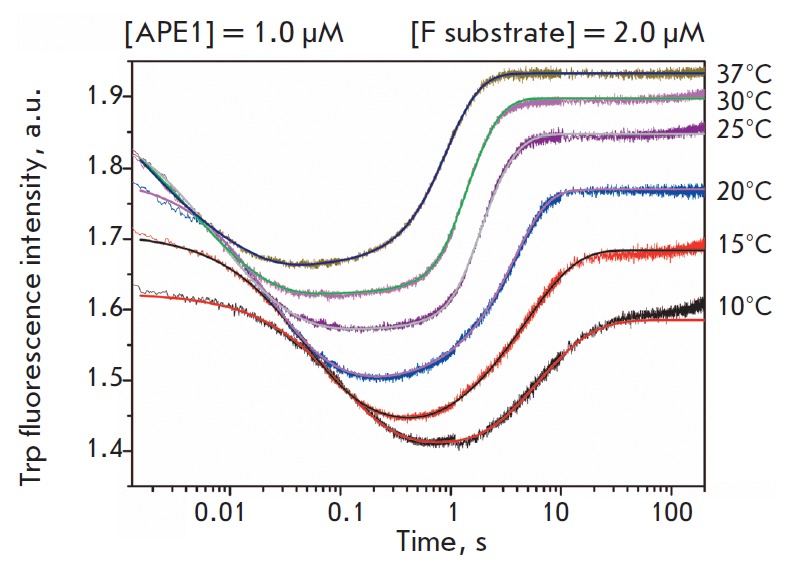
Changes in the Trp fluorescence intensity during interaction between APE1 and the F substrate at different temperatures. [APE1] = 1.0 μM, [F substrate] = 2.0 μM.

To clarify the nature of the processes occurring during sequential stages of F site recognition in the DNA-substrate complex, catalysis, and enzyme-product complex dissociation, we conducted a stepwise thermodynamic analysis of the interaction between APE1 and the F substrate. Stopped-flow measurements of the Trp fluorescence intensity provided kinetic curves characterizing the interaction between APE1 and the 17- mer F substrate at one enzyme turnover conditions and temperature of 10 to 37°C (*Fig. 3*). It is seen that the interaction between APE1 and the F substrate leads to multiphase changes in the Trp fluorescence intensity. According to the previously obtained data [14, 15], a decrease in the fluorescence intensity in the initial part of the kinetic curves characterizes the formation of a catalytically competent complex. The catalytic reaction step leading to the formation of products and subsequent dissociation of the enzyme-product complex is accompanied by an increase in the Trp fluorescence intensity at longer times ( > 1 s). As is evident from the kinetic curves (*Fig. 4*), both phases of the changes in the fluorescence intensity are temperature-dependent.

An analysis of the kinetic curves of the protein fluorescence intensity demonstrated that the minimum kinetic mechanism of the interaction between APE1 and the DNA substrate containing the F site as damage involves a two-step equilibrium binding, irreversible formation of the enzyme-product complex, and equilibrium dissociation of the complex. As previously [14-16], the mechanism is described by the *Scheme*

The rate constants of the forward and reverse reactions that describe the APE1-DNA substrate interaction at different temperatures were calculated by a nonlinear regression, including numerical integration of differential equations related to the *Scheme*, as described previously [28, 29]. The resulting rate constants were used to determine the equilibrium constants *K*i and *K*p (*Table 1*).

**Table 1:** Rate constants for individual steps of the interaction between APE1 and the F substrate under BER conditions and dissociation constants of the enzyme-product complex.

Temperature Constant	10°C	15°C	20°C	25°C	30°C	37°C
k_1_, M^-1^c^-1^	(5.1 ± 2.1) × 10^6^	(16.0 ± 3.4) × 10^6^	(46.0 ± 12.0) × 10^6^	(100 ± 12) × 10^6^	(190 ± 32) × 10^6^	(520 ± 20) × 10^6^
k^-1^, c^-1^	3.3 ± 0.4	7.3 ± 3.6	12.0 ± 5.1	11.0 ± 2.5	19.0 ± 5.2	47.0 ± 13.0
K_1_*, M	0.65 × 10^-6^	0.47 × 10^-6^	0.26 × 10^-6^	0.11 × 10^-6^	0.10 × 10^-6^	0.09 × 10^-6^
k_2_, c^-1^	4.2 ± 2.6	3.7 ± 1.2	8.2 ± 3.9	8.8 ± 4.4	15.0 ± 1.8	24.0 ± 9.0
k_-2_, c^-1^	5.7 ± 1.9	5.5 ± 2.9	19.0 ± 3.7	27.0 ± 4.2	40.0 ± 6.6	93.0 ± 17.4
K_2_	1.27	0.51	0.68	0.81	0.79	0.52
k_cat_, c^-1^	1.4 ± 0.6	2.0 ± 0.7	2.5 ± 1.2	4.6 ± 2.2	6.6 ± 2.2	9.2 ± 1.0
K_p_, M	(13.5 ± 3.9) × 10^-6^	(10.6 ± 1.9) × 10^-6^	(7.2 ± 1.8) × 10^-6^	(6.6 ± 2.3) × 10^-6^	(6.9 ± 1.2) × 10^-6^	(4.2 ± 0.6) × 10^-6^

*Equilibrium association constants were calculated using the formula *K*_i_*= k_–i_/k_i_.*

**Fig. 5:**
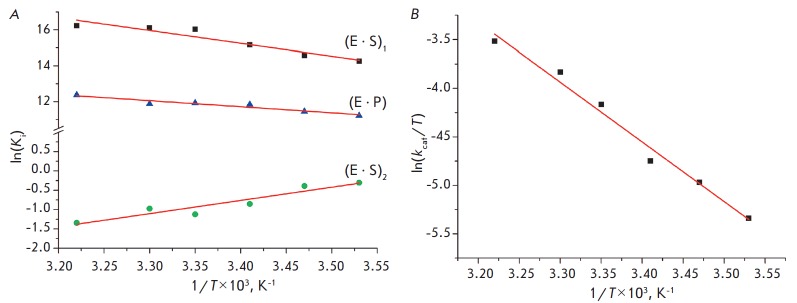
Analysis of the temperature dependence of ln(*K*_i_) (A) and ln(*k*_cat_/T) (B).

As shown in *Fig. 5*, the ln(*K*_i_) and ln(*k*_cat_/T) vs 1/T dependences are linear, which enables calculation of thermodynamic parameters for equilibrium steps using the van’t Hoff equation (1), as well as parameters of the transition state in the catalytic step using the Eyring equation (2) (*Table 2*).

According to the obtained data, the formation of the primary enzyme-substrate complex (the first step in the *Scheme*) is characterized by a positive standard enthalpy value (14.3 kcal/mol) and a positive entropy value (79.0 cal/(mol×K)). An increase in entropy during interaction between DNA-binding proteins and DNA is known to be usually due to two factors: desolvation of polar groups at the DNA-protein interface [30] and removal of highly ordered molecules of “crystalline water” from the DNA grooves [31]. It may be assumed that the bonds between amino acid residues of the DNA-binding site and the DNA duplex form at this stage. The interaction between DNA duplex phosphate groups situated upstream and downstream of the F site and Arg73, Ala74, Lys78, Trp280, Asn222, Asn226, and Asn229 residues is of special interest (*Fig. 1*). Furthermore, incorporation of the Arg177 residue into the DNA duplex on the major groove side and formation of a hydrogen bond with a phosphate group located downstream of the F site may occur at this moment. The Met270 residue is incorporated into the DNA duplex on the minor groove side and may also displace “crystalline” water. Previously, studies of *E. coli *Fpg [28] and human OGG1 [29] DNA-glycosylases, which belong to different structural classes and, consequently, interact with DNA through contacts of different nature, demonstrated that the steps of enzyme-substrate complex formation and isomerization of the complex into a catalytically competent state are characterized by a significant increase in entropy that is apparently caused by desolvation of the interacting protein and DNA surfaces.

**Table 2:** Thermodynamic parameters of the interaction between APE1 and F-substrate.

Parameter Step (number)	ΔG°_i,298_, kcal/mol	ΔH°^i^, kcal/mol	ΔS°_i_, cal/(mol×K)
Primary DNA binding (1)	–9.2	14.3 ± 2.2	79.0 ± 7.6
Specific recognition of the F site (2)	0.5	–6.8 ± 1.2	–24.6 ± 4.0
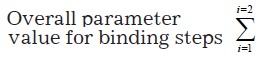	–8.7	7.5 ± 3.4	54.4 ± 11.7
Transition state of the catalytic step (3)	16.6	12.2 ± 0.8	–14.8 ± 2.8
			
Enzyme-product complex formation (4)	–7.0	6.8 ± 1.0	46.6 ± 3.5

The second stage of the interaction between APE1 and the F substrate is a specific rearrangement of the (E•S)_1_ complex and is characterized by negative changes in both enthalpy (ΔH°_2_ = –6.8 kcal/mol) and entropy (ΔS°_2_ = –24.6 cal/(mol×K)). The negative ΔH°_2_ value indicates stabilization of the complex during formation of new, energetically favorable bonds among interacting atoms, while the negative ΔS°_2_ value suggests an increase in the rigidity of the complex; i.e. a reduction in its internal degrees of freedom. This step probably involves flipping the F site into the enzyme active site and stabilizing this state by Arg177 and Met270 residues that are inserted into the major and minor DNA grooves, respectively. Furthermore, bonds between a phosphate group located upstream of the F site (*Fig. 1*) and the Asn174, Asn212, and His309 residues and the Mg^2+^ ion that are located in the enzyme active site may form at this moment.

Activation enthalpy (ΔH^‡^) and entropy (ΔS^‡^) for the transition complex formation were calculated for the third catalytic step. The resulting activation enthalpy value is 12.2 kcal/mol. It should be noted that this value is related to the step of phosphodiester bond hydrolysis by the APE1 enzyme and lies within the range of 6.0–18.6 kcal/mol obtained previously for the catalytic steps of N-glycosidic bond cleavage and β-elimination of phosphate groups by Fpg and hOGG1 DNA glycosylases [28, 29].

The thermodynamic parameters of the complex formation between APE1 and AP-containing DNA were obtained previously using SPR, i.e. under heterophase conditions, for a catalytically inactive enzyme in the absence of Mg^2+^ ions [17]. The approach used in the present study [28, 29] enables the calculation of thermodynamic data for processes occurring in an aqueous solution, i.e. under homophase conditions, and involving catalytically active forms of enzymes, including transient enzyme-substrate intermediates.

Interestingly, the thermodynamic parameters for the step of the complex formation between the enzyme and a reaction product correlate with those of the primary complex formation. Similarly to the first step, this process is characterized by positive standard enthalpy and entropy changes (6.8 kcal/mol and 46.6 cal/(mol×K), respectively). This indicates that the thermodynamic parameters of this step are largely determined by the same interactions that occur at the first step of APE1 binding to the DNA substrate – nonspecific contacts between the DNA-binding site and the ribose-phosphate backbone of the DNA duplex. However, the enzyme-product complex (E•P) may be considered a true non-specific complex, while the formation of the primary complex (E•S)_1_ in the case of a short DNA substrate involves some elements of specific recognition of the F site. Therefore, the (E•S)_1_ complex formation is energetically more favorable compared to the E•P complex (ΔΔG°_298_ = –2.2 kcal/mol, ΔΔH° = 7.5 kcal/mol, ΔΔS° = 32.4 cal/(mol×K)).

Thus, we obtained the thermodynamic parameters of conformational APE1 rearrangements associated with specific recognition of a damaged DNA fragment and the catalytic step. These findings led to a conclusion on the molecular nature of the individual steps of the kinetic mechanisms that describe the enzyme function.

## Abbreviations

APE1: AP endonuclease
BER: base excision repair
AP site: apurinic/apyrimidinic site
F site: (3-hydroxy-tetrahydrofuran-2-yl) methyl phosphate
